# Autophagy-Related Proteins Are Differentially Expressed in Adrenal Cortical Tumor/Pheochromocytoma and Associated with Patient Prognosis

**DOI:** 10.3390/ijms221910490

**Published:** 2021-09-28

**Authors:** Hye Min Kim, Ja Seung Koo

**Affiliations:** Department of Pathology, Yonsei University College of Medicine, Seoul 03722, Korea; pinkmin15@yuhs.ac

**Keywords:** autophagy, subtype, adrenal gland tumor, stroma, pathology

## Abstract

The aim of this research was to evaluate the expression and concomitant implications of LC3A, LC3B, beclin-1, and p62, which are key components of autophagy in human adrenal gland tumors. Tissue microarray was made for 321 cases of adrenal gland tumor (adrenal cortical adenoma (ACA): 115, adrenal cortical carcinoma (ACC): 17, and pheochromocytoma (PCC): 189). Immunohistochemical staining was performed for beclin-1, p62, LC3A, and LC3B, and the results were compared with the patients’ clinicopathologic parameters. LC3A, LC3B, beclin-1, and LC3B isolated single positive cells (ISPC) positivity rates were higher in PCC than in adrenal cortical tumor (ACT), whereas p62 positivity was lower in PCC than in ACT. The proportion of positive LC3B (ISPC) was higher in ACC than in ACA. In addition, the proportion of cells positive for p62 and LC3B (ISPC) was significantly higher in PCCs with a GAPP score of ≥3. In univariate Cox analysis, p62 positivity (*p* = 0.014) and the presence of p62 (ISPC) (*p* = 0.001) were associated with shorter disease-free survival in PCC. Moreover, p62 positivity was predictive of shorter overall survival (OS) in patients with PCC by multivariate analysis (relative risk, 6.240; 95% CI, 1.434–27.15; *p* = 0.015). Differences were found in the expression of autophagy-related proteins according to adrenal gland tumor types. Compared to ACT, the proportion of LC3A, LC3B, beclin-1, and LC3B (ISPC) positivity was higher in PCC, whereas p62 positivity was lower. Similarly, p62 positivity in PCC was associated with patient prognosis of OS.

## 1. Introduction

Adrenal gland tumors are typically categorized into adrenal cortical tumors (ACTs), originating from the adrenal cortex, and pheochromocytomas (PCCs), which occur in the adrenal medulla. ACT is a relatively rare tumor and is classified into adrenal cortical adenoma (ACA) and adrenal cortical carcinoma (ACC), with histological differential diagnosis between the two being a difficult task. ACC is a particularly rare tumor associated with extremely poor prognosis due to the absence of effective target modalities and the fact that most of the tumor biology is still largely unknown. However, the distinction between benign and malignant PCC based on histological findings is quite challenging, and malignant PCC can be confirmed only when distant metastasis occurs. Consequently, there is an unmet clinical need to discover histopathologic features that could predict prognosis in adrenal gland tumors.

Autophagy is defined as the physiologic process of lysosomal degradation of cellular components. Autophagy plays an important homeostatic role by removing unnecessary and/or dysfunctional cellular components and reusing essential cellular components [1,2,3,4]. Among the various indicators to evaluate autophagy process, protein markers used to evaluate the activity of autophagy contain: beclin-1 [5,6,7,8], which takes part in nucleation; LC3 [9,10,11], which contributes to the formation of the autophagosome by participating in elongation; p62 [12,13], a scaffold protein that transports ubiquitinated proteins to the autophagosome; and BNIP-3 [14], a mitochondrial autophagy (mitophagy) regulator. Increasing results indicate that autophagy is related to tumor biology and may act as a tumor behavior. In the case of high-grade malignant tumors that are characterized by increased metabolic demand, angiogenesis and/or aerobic glycolysis alone may not be able to meet the metabolic demand of tumor cells. In these circumstances, malignant cells derive energy by recycling cytoplasmic components through autophagy as an alternative [15,16]. Meanwhile, unrestrained autophagy can cause progressive consumption of cellular constituents, leading to cell death [17,18]. Therefore, autophagy can theoretically contribute to both tumor progression and suppression; however, there is a lack of detailed research about the expression of autophagy-related proteins in adrenal gland tumors. The aim of this study was to investigate the expression and implication of key autophagy components, LC3A, LC3B, beclin-1, and p62, in human adrenal gland tumors.

## 2. Materials and Methods

### 2.1. Patient Selection

Patients at Severance Hospital diagnosed with ACT or PCC after completing surgical removal between January 2000 and December 2013 were consecutively included. Surgical specimens were reviewed retrospectively by an expert endocrine pathologist (Koo JS and Kim HM), and histologic evaluation was conducted using H&E–stained slides. Clinical and pathologic data were also retrospectively collected from the patients’ electronic medical records, which included sex, age at diagnosis, local recurrence, distant metastasis, patient death, and the length of follow up. This study was approved by the Institutional Review Board of Yonsei University Severance Hospital (9 March 2021; 4-2021-0028), which exempted informed consent from patients.

### 2.2. In Silico Analysis

We searched the gene expression database of normal and tumor tissues (GENT2), a web-accessible database, to compare beclin-1 and LC3A expression patterns in adrenal gland tumor and normal tissues in 20 March 2021 (http://gent2.appex.kr/gent2/). Additionally, we used the web-accessible database cBioPortal in 20 March 2021 (http://www.cbioportal.org) to investigate *MAP1LC3A* gene alterations in adrenal gland tumor tissues (Figure S1).

### 2.3. Tissue Microarray

For the creation of tissue microarray, typical areas on H&E slides were selected and the corresponding area was marked on the corresponding paraffin block. Three-millimeter cores were selected from the marked area and placed into a 6 × 5 recipient block. Two tissue cores were selected to avoid selection bias. Each core was randomly assigned into a unique location number linked to clinical and pathologic data.

### 2.4. Immunohistochemistry

Commercial antibodies used in this study are itemized in Table S1. Immunohistochemical staining was conducted with formalin-fixed, paraffin-embedded tissue sections by an automatic staining system (Benchmark XT, Ventana Medical System, Tucson, AZ, USA) according to the manufacturer’s instructions. Negative control tissue was stained in the absence of primary antibodies and the positive control sample was selected as recommended and counterstained with Harris hematoxylin. 

### 2.5. Interpretation of Immunohistochemical Staining

Beclin-1, p62, LC3A, and LC3B stained slides were identified via light microscopy and were assessed semi-quantitatively across the entire tumor area as previously described [19]. Briefly, the staining score was determined as 0: negative or weak expression in less than 1%, 1: focal expression in 1–10%, 2: positive staining in 11–50%, and 3: positive staining in 51–100%. We defined scores of 0–1 as negative, and a score of 2 and 3 as positive.

### 2.6. Statistical Analysis

Clinical, pathological, and immunohistochemical results were analyzed by SPSS, version 21.0 (released 2012; IBM Corp., Armonk, NY, USA). To determine statistical significance, chi-squared and Fisher’s exact tests were adopted for categorical variables and a Student’s t-test was used for continuous variables. Kaplan-Meier curves were used to evaluate disease-free survival and overall survival. The difference of survival was determined by log-rank statistics. Multivariate analysis using the Cox proportional hazards model was applied. Statistical significance was determined as *p* < 0.05.

## 3. Results

### 3.1. Clinicopathologic Characteristics of Patients

For ACT, a total of 132 cases, 115 (87.1%) ACA and 17 (12.9%) ACC, were included. The clinicopathologic features of ACT patients are presented in Table S2. The clinical factors with significant differences between ACA and ACC were age (*p* = 0.048) and tumor size (*p* < 0.001). Factors included in the Weiss system were also significantly different between ACA and ACC (all *p* < 0.001, Table S2). On the other hand, the clinicopathologic features of 189 cases of PCC are presented in Table S3.

### 3.2. Expression of Autophagy-Related Proteins in ACT and PCC

**Figure 1:**
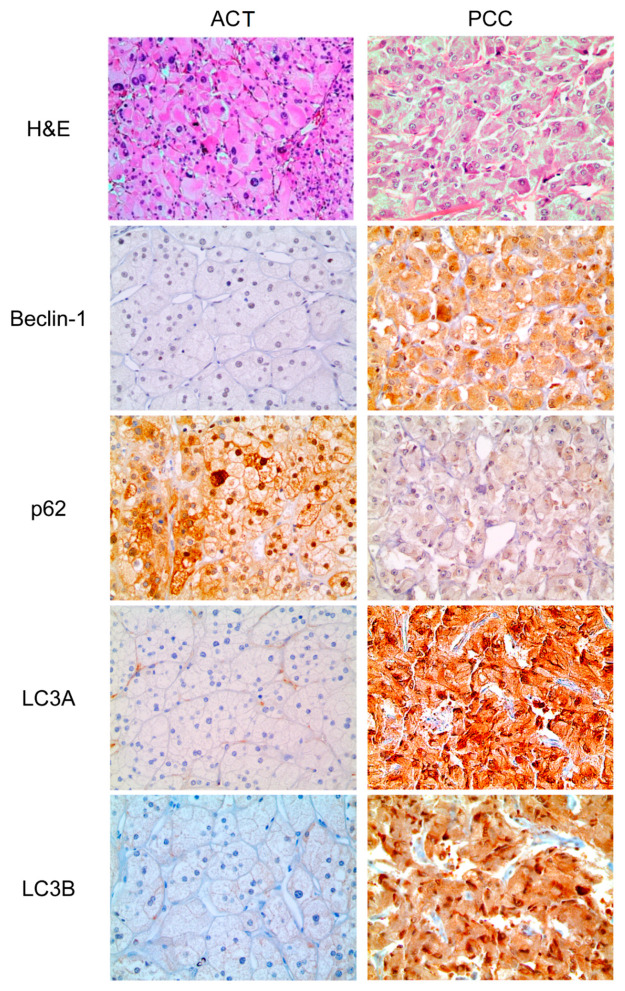
Expression of autophagy-related proteins in adrenal cortical tumor and pheochromocytoma. LC3A, LC3B, beclin-1, and LC3B (ISPC) positivity rates were higher in PCC than in ACT, whereas p62 positivity was lower in PCC.

**Figure 2:**
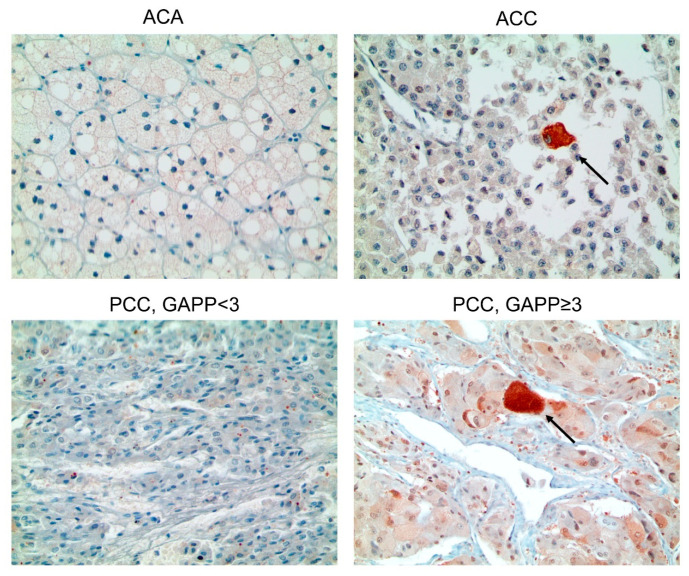
Isolated single positive cells (ISPC) of p62 and LC3B stain in adrenal tumors. The ISPC proportion of LC3B was higher in ACC than in ACA. The ISPC proportions of p62 and LC3B were significantly higher in PCCs with a GAPP score of ≥3 than in PCCs with a GAPP score of <3. Arrows indicate ISPC.

In some cases, isolated single positive cells (ISPC) were observed for p62 and LC3B. Strong cytoplasmic expression was detected in one cell (Figure S2). Statistically significant differences were noted between ACT and PCC. The proportion of beclin-1 (*p* < 0.001), LC3A (*p* < 0.001), LC3B (*p* < 0.001), and LC3B (ISPC) (*p* = 0.010) positivity were higher in PCC than in ACT, whereas the proportion of p62 positive cells was lower in PCC than in ACT (*p* = 0.006) (Table 1 and Figure 1). A comparison of autophagy-related protein expression between ACA and ACC demonstrated that the proportion of positive LC3B (ISPC) was higher in ACC than in ACA (*p* = 0.027, Table 2 and Figure 2). The proportions of cells positive for p62 (*p* = 0.017) and LC3B (ISPC) (*p* = 0.013) were significantly higher in PCCs, with a GAPP score of ≥3 (Table 3 and Figure 2).

### 3.3. Presence/Absence of Clinicopathologic Factors in PCC/ACT and Expression of Autophagy-Related Proteins

**Figure 3:**
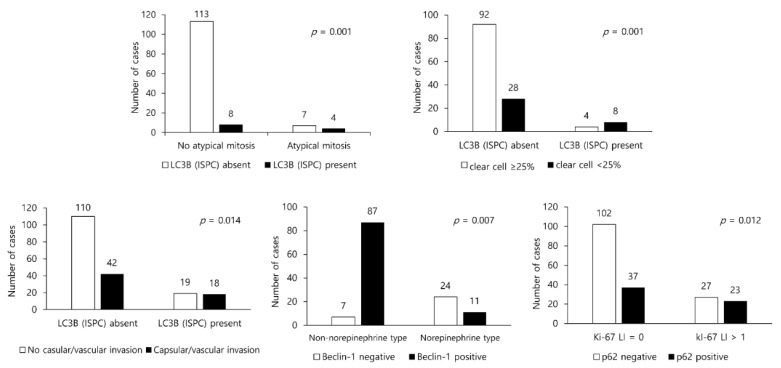
The presence/absence of clinicopathologic factors and the expression of autophagy-related proteins in adrenal cortical tumor and pheochromocytoma.

In ACT, LC3B (ISPC) positivity was associated with atypical mitosis (*p* = 0.001) and clear cell proportion (*p* = 0.001). In the presence of positive LC3B (ISPC), the proportion of atypical mitosis and the incidence of clear cells being < 25% was significantly higher (Figure 3). In PCC, the number of cells positive for LC3B (ISPC) was significantly associated with capsular/vascular invasion (*p* = 0.014). Furthermore, beclin-1 positivity was associated with norepinephrine type (*p* = 0.007), whereas p62 positivity was related to Ki-67 labeling index (L.I.) >1 (*p* = 0.012). The capsular/vascular invasion ratio was high in the presence of positive LC3B (ISPC). Meanwhile, the ratio of non-norepinephrine type was higher when beclin-1 expression was positive, and the ratio of Ki-67 L.I. > 1 was found to be higher in the case of cells positive for p62 expression (Figure 3).

### 3.4. Impact of the Expression of Autophagy-Related Proteins on Patient Prognosis in PCC/ACT

**Figure 4:**
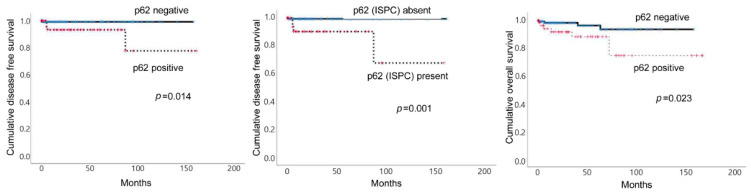
Impact of the expression of autophagy-related proteins on prognosis in pheochromocytoma. The p62 (left figure) and p62 (ISPC) (middle figure) positivity was associated with shorter disease-free survival in PCC, whereas p62 positivity was also associated with shorter overall survival (right figure).

In the univariate analysis, p62 positivity (*p* = 0.014) and the presence of p62 (ISPC) positive cells (*p* = 0.001) were associated with shorter disease-free survival (DFS) in PCC. Moreover, p62 positivity (*p* = 0.023) was associated with shorter overall survival (OS) (Table 4 and Figure 4). The multivariate Cox analysis revealed that none of the autophagy-related proteins were significantly associated with DFS, but p62 positivity was predictive of shorter OS (relative risk, 6.240; 95% CI, 1.434-27.15; *p* = 0.015, Table 5). There was no significant relationship between the expression of the assessed autophagy-related proteins and patient prognosis in ACT (Table 6).

## 4. Discussion

In this study, we evaluated the expression status of autophagy-related proteins in adrenal gland tumors. Autophagy is regarded as playing a crucial role in the adrenal gland during physiologic states, since it regulates the growth of adrenal cells in the zona fasciculata [20]. We found that, compared to ACT, the proportion of cells positive for beclin-1, LC3A, LC3B, and LC3B (ISPC) was higher in PCC, whereas p62 positivity was lower in PCC. Furthermore, among these proteins, p62 positivity was revealed to have prognostic significance in PCC, suggesting that identification of proteins involved in autophagy could have clinical relevance. This differential protein expression, according to the origin of adrenal gland tumors, could be relevant to a previous study that reported that autophagy exerts hormone-producing modulatory effects in steroid-secreting cells in the adrenal cortex, but not in the adrenal medulla, wherein the former exhibited characteristics similar to those of Leydig cells in the testis [21]. 

In the present study, the proportion of cells positive for LC3B (ISPC) was higher in ACC than ACA. Additionally, in PCC, it was associated with a GAPP score of over 3, the presence of atypical mitosis, and capsular/vascular invasion, all of which are clinical features indicating poor patient prognosis. In general, autophagy is understood as a cellular mechanism that degrades intracellular components and produces amino acids, nucleotides, fatty acids, sugars, and ATP by recycling proteins and organelles under oxidative stress [22,23]. However, newly emerging literature suggests that autophagy facilitates tumor progression, survival, and colonization in distant organs [24,25,26,27,28]. The recycling of intracellular components to supply metabolic substrates to overcome the stressful conditions of hypoxia or malnutrition in cancers is a putative explanation for the abovementioned phenomena. It was reported that the expression of autophagy-related proteins was higher in breast cancer with brain metastasis than in a primary breast tumor, corroborating our hypothesis [29]. Moreover, beclin-1 was reported as a marker of gastric carcinogenesis, aggressiveness, and a prognostic marker [30]. Our study also found that aggressive features were related to the expression of autophagy-related proteins. Differently, autophagy could influence patient prognosis via affecting response to chemotherapy [31]. Interestingly, it was found that p62 positivity was related to shorter DFS and OS in PCC. In a previous study, lithium increased autophagy in PC12 cells, resulting in overgrowth of PCC cells, suggesting the possibility that autophagy-related proteins could be prognostic biomarkers in adrenal gland tumors [32]. 

As shown previously, autophagy is known to act as a tumor enhancer and/or tumor suppressor in various cancers, is also pathogenically implicated in tumor cell growth in adrenal gland tumors [30]. Nevertheless, because the expression of autophagy-related proteins in adrenal gland tumors was not reported in the existing literature, a direct comparison of our findings to a previous study could not be conducted.

Another potential clinical implication of this study is that it paved the way for compounds targeting autophagy as promising therapeutic agents in patients with adrenal gland tumors [33,34,35,36]. A study using rosiglitazone (RGZ), a PPAR-γ agonist, showed that RGZ was capable of activating the AMPK pathway, leading to enhanced autophagy, reactive oxygen species formation, as well as upregulating beclin-1 and LAMP-1, a protein involved in the process of autophagy to induce autophagic cell death in ACC cell lines [37]. However, a contradictory result was reported in PCC, as the induction of autophagy in the rat PCC cell line, PC12, improved cell survival [32,38]. Sunitinib is a tyrosine kinase inhibitor that induces apoptosis and autophagy in PC12 cells by direct inhibition of mTOR [39]. Inhibition of the autophagy process promoted the anti-proliferative and apoptotic effect of sunitinib, highlighting an inverse effect as compared with ACC [38]. Further, a study using SW13 cells, which is an ACC cell line, demonstrated that RGZ does not exert any effect on autophagy; instead, it accompanies the process of cell cycle dysregulation and the inhibition of cell growth [37], emphasizing that the effect of autophagy is complex and could largely differ based on the underlying tumor microenvironment. Notably, as there are increasing attempts to develop therapeutic agents targeting autophagy in cancers [33,34,35,36], it may be possible that these drugs could be used in the treatment of adrenal gland tumors, the possibility and efficacy of which will be elucidated by further in vitro and in vivo studies in the future.

The limitation of this study is that the number of patients with ACC was small, and only a small proportion of patients with PCC (8 among the 189 patients) had undergone chemotherapy. Thus, the effect of autophagy-related protein expression in requiring chemotherapy could not be evaluated. In addition, immunohistochemical staining may not be sufficient to precisely evaluate the activity of autophagy. Given that the evaluation of autophagy was only performed using paraffin blocks of adrenal gland tumors, a detailed investigation utilizing autophagy flux, which enables assessment of the complex, dynamic, and the multi-sequential process of autophagy, as well as in vitro investigations (i.e., immunofluorescence and cell line studies) is warranted to provide better insights into the role of autophagy in adrenal gland tumors.

## 5. Conclusions

The expression of autophagy-related proteins was significantly different between PCC and ACT. In particular, the expressions of these proteins were associated with aggressive clinical features and patient prognosis, suggesting that the estimation of these proteins may be useful in the discrimination of adrenal gland tumors and the identification of patients with poor prognosis.

## Figures and Tables

**Table 1:** Expression of autophagy-related proteins in adrenal cortical tumor and pheochromocytoma.

Parameters	Total n = 321 (%)	Adrenal Cortical Tumor n = 132 (%)	Pheochromocytoma n = 189 (%)	*p*-Value
Beclin-1				<0.001
Negative	218 (67.9)	127 (96.2)	91 (48.1)	
Positive	103 (32.1)	5 (3.8)	98 (51.9)	
p62				0.006
Negative	199 (62.0)	70 (53.0)	129 (68.3)	
Positive	122 (38.0)	62 (47.0)	60 (31.7)	
p62 (ISPC)				0.128
Absent	238 (74.1)	92 (69.7)	146 (77.2)	
Present	83 (25.9)	40 (30.3)	43 (22.8)	
LC3A				<0.001
Negative	221 (68.8)	132 (100.0)	89 (47.1)	
Positive	100 (31.2)	0 (0.0)	100 (52.9)	
LC3B				<0.001
Negative	243 (75.7)	116 (87.9)	127 (67.2)	
Positive	78 (24.3)	16 (12.1)	62 (32.8)	
LC3B (ISPC)				0.010
Absent	272 (84.7)	120 (90.9)	152 (80.4)	
Present	49 (15.3)	12 (9.1)	37 (19.6)	

**Table 2:** Expression of autophagy-related proteins in adrenal cortical adenoma and adrenal cortical carcinoma.

Parameters	Total n = 132 (%)	Adrenal Cortical Adenoma n = 115 (%)	Adrenal Cortical Carcinoma n = 17 (%)	*p*-Value
Beclin-1				0.381
Negative	127 (96.2)	110 (95.7)	17 (100.0)	
Positive	5 (3.8)	5 (4.3)	0 (0.0)	
p62				0.294
Negative	70 (53.0)	63 (54.8)	7 (41.2)	
Positive	62 (47.0)	52 (45.2)	10 (58.8)	
p62 (ISPC)				0.107
Absent	92 (69.7)	83 (72.2)	9 (52.9)	
Present	40 (30.3)	32 (27.8)	8 (47.1)	
LC3A				n/a
Negative	132 (100.0)	115 (100.0)	17 (100.0)	
Positive	0 (0.0)	0 (0.0)	0 (0.0)	
LC3B				0.101
Negative	116 (87.9)	99 (86.1)	17 (100.0)	
Positive	16 (12.1)	16 (13.9)	0 (0.0)	
LC3B (ISPC)				0.027
Absent	120 (90.9)	107 (93.0)	13 (76.5)	
Present	12 (9.1)	8 (7.0)	4 (23.5)	

**Table 3:** Expression of autophagy-related proteins in pheochromocytoma according to GAPP score.

Parameters	Total n = 189 (%)	Pheochromocytoma		*p*-Value
		GAPP Score < 3 n = 138 (%)	GAPP Score ≥ 3 n = 51 (%)	
Beclin-1				0.884
Negative	91 (48.1)	66 (47.8)	25 (49.0)	
Positive	98 (51.9)	72 (52.2)	26 (51.0)	
p62				0.017
Negative	129 (68.3)	101 (73.2)	28 (54.9)	
Positive	60 (31.7)	37 (26.8)	23 (45.1)	
p62 (ISPC)				0.349
Absent	146 (77.2)	109 (79.0)	37 (72.5)	
Present	43 (22.8)	29 (21.0)	14 (27.5)	
LC3A				0.747
Negative	89 (47.1)	64 (46.4)	25 (49.0)	
Positive	100 (52.9)	74 (53.6)	26 (51.0)	
LC3B				0.254
Negative	127 (67.2)	96 (69.6)	31 (60.8)	
Positive	62 (32.8)	42 (30.4)	20 (39.2)	
LC3B (ISPC)				0.013
Absent	152 (80.4)	117 (84.8)	35 (68.6)	
Present	37 (19.6)	21 (15.2)	16 (31.4)	

**Table 4:** Univariate analysis of the impact of expression of autophagy-related proteins in pheochromocytoma on disease-free survival and overall survival by the log-rank test.

Parameter	Number of Patients /Recurrence/Death	Disease-Free Survival		Overall Survival	
		Mean Survival (95% CI) Months	*p*-Value	Mean Survival (95% CI) Months	*p*-Value
Beclin-1			0.655		0.112
Negative	91/2/8	150 (139–161)		142 (126–158)	
Positive	97/3/3	154 (148–161)		159 (150–167)	
p62			0.014		0.023
Negative	128/1/4	156 (154–159)		150 (142–157)	
Positive	60/4/7	139 (115–162)		136 (114–157)	
p62 (ISPC)			0.001		0.910
Absent	145/1/9	159 (157–162)		144 (134–155)	
Present	43/4/2	127 (96–157)		157 (144–170)	
LC3A			0.657		0.435
Negative	89/3/4	146 (136–157)		154 (142–166)	
Positive	99/2/7	157 (151–162)		142 (129–155)	
LC3B			0.529		0.220
Negative	126/4/5	149 (140–158)		155 (145–165)	
Positive	62/1/6	157 (151–164)		138 (121–155)	
LC3B (ISPC)			0.234		0.410
Absent	151/3/10	154 (147–162)		148 (137–160)	
Present	37/2/1	145 (129–162)		151 (139–163)	

**Table 5:** Multivariate analysis of disease-free survival and overall-survival of patients with pheochromocytoma.

Included Factor	Disease-Free Survival			Overall Survival		
	Hazard Ratio	95% CI	*p*-Value	Hazard Ratio	95% CI	*p*-Value
Histologic pattern			0.738			0.720
Zellballen vs. Non-Zellballen	1.491	0.143–15,351		0.737	0.139–3.912	
Cellularity			0.290			0.504
Low, moderate vs. High	3.093	0.382–25.02		1.827	0.312–10.71	
Vascular and/or capsular invasion			0.524			0.183
Absent vs. Present	2.125	0.210–21.55		2.854	0.610–13.34	
Ki-67 labeling index (%)			0.923			0.112
<1 vs. ≥1	1.598	0.000–21,497		0.124	0.010–1.623	
GAPP score			0.892			0.061
0–2 vs. 3–10	1.949	0.000–29,027		13.906	0.884–218.8	
p62			0.420			0.015
Negative vs. Positive	2.945	0.123–40.72		6.240	1.434–27.15	
p62 (ISPC)			0.081			0.432
Absent vs. Present	8.143	0.771–85.95		0.522	0.103–2.638	

**Table 6:** Univariate analysis of the impact of expression of autophagy-related proteins in adrenal cortical tumors on disease-free survival and overall survival by the log-rank test.

Parameter	Number of Patients /Recurrence/Death	Disease-Free Survival		Overall Survival	
		Mean Survival (95% CI) Months	*p*-Value	Mean Survival (95% CI) Months	*p*-Value
Beclin-1			n/a		n/a
Negative	127/3/9	n/a		n/a	
Positive	5/0/0	n/a		n/a	
p62			0.446		0.056
Negative	70/1/2	107 (104–110)		106 (102–110)	
Positive	62/2/7	115 (110–120)		106 (97–115)	
p62 (ISPC)			0.138		0.322
Absent	92/1/5	107 (105–109)		103 (99–108)	
Present	40/2/4	113 (105–121)		107 (96–118)	
LC3A			n/a		n/a
Negative	132/3/9	n/a		n/a	
Positive	0/0/0	n/a		n/a	
LC3B			n/a		n/a
Negative	116/3/9	n/a		n/a	
Positive	16/0/0	n/a		n/a	
LC3B (ISPC)			0.094		0.157
Absent	120/2/7	107 (105–109)		103 (98–107)	
Present	12/1/2	108 (88–128)		100 (77–123)	

n/a: not available.

## Data Availability

The datasets used to support the findings of this study are available from the corresponding author upon request.

## Supplementary Materials

The following are available online at https://www.mdpi.com/article/10.3390/ijms221910490/s1.
