# Supplementary material for: Autophagy-Related Proteins Are Differentially Expressed in Adrenal Cortical Tumor/Pheochromocytoma and Associated with Patient Prognosis
Source: Int J Mol Sci. 2021 Sep 28;22(19):10490. doi: 10.3390/ijms221910490 (PMC8508962; doi:10.3390/ijms221910490)

Supplementary Table S1. Source, clone, and dilution of used antibodies

Antibody	Clone	Dilution	Company
Beclin-1	Polyclonal	1:100	Abcam, Cambridge, UK
p62	SQSTM1	1:100	Abcam, Cambridge, UK
LC3A	EP1528Y	1:100	Abcam, Cambridge, UK
LC3B	Polyclonal	1:100	Abcam, Cambridge, UK

Supplementary Table S2. Basal characteristics of adrenal cortical tumor

Parameters	Total<br>N=132 (%)	Adrenal<br>cortical adenoma<br>n=115 (%)	Adrenal<br>cortical carcinoma<br>n=17 (%)	p-value
Age<br>(year,<br>mean±SD)	47.5±145	48.4±12.2	41.0±25.1	**0.048**
Sex				0.107
Male	40 (30.3)	32 (27.8)	8 (47.1)	
Female	92 (69.7)	83 (72.2)	9 (52.9)	
Tumor size<br>(cm, mean±SD)	3.6±3.7	2.5±1.3	10.9±5.8	**<0.001**
Fuhrman grade				**<0.001**
1-2	106 (80.3)	102 (88.7)	4 (23.5)	
3-4	26 (19.7)	13 (11.3)	13 (76.5)	
Mitosis				**<0.001**
≤5/50HFPs	122 (92.4)	115 (100.0)	7 (41.2)	
>5/50HFPs	10 (7.6)	0 (0.0)	10 (58.8)	
Atypical mitosis				**<0.001**
Absent	121 (91.7)	114 (99.1)	7 (41.2)	
Present	11 (8.3)	1 (0.9)	10 (58.8)	
Clear<br>cell proportion				**<0.001**
≥25%	96 (72.7)	95 (82.6)	1 (5.9)	
<25%	36 (27.3)	20 (17.4)	16 (94.1)	
Diffuse<br>architecture				**<0.001**
Absent	117 (88.6)	111 (96.5)	6 (35.3)	
Present	15 (11.4)	4 (3.5)	11 (64.7)	
Necrosis				**<0.001**
Absent	113 (85.6)	113 (98.3)	0 (0.0)	

Present	19 (14.4)	2 (1.7)	17 (100.0)	
Venous invasion				**<0.001**
Absent	126 (95.5)	115 (100.0)	11 (64.7)	
Present	6 (4.5)	0 (0.0)	6 (35.3)	
Sinusoidal invasion				**<0.001**
Absent	126 (95.5)	115 (100.0)	11 (64.7)	
Present	6 (4.5)	0 (0.0)	6 (35.3)	
Capsular invasion				**<0.001**
Absent	117 (88.6)	111 (96.5)	6 (35.3)	
Present	15 (11.4)	4 (3.5)	11 (64.7)	
Weiss total score				**<0.001**
< 4	117 (88.6)	115 (100.0)	2 (11.8)*	
≥ 4	15 (11.4)	0 (0.0)	15 (88.2)	
Recurrence	3 (2.3)	0 (0.0)	3 (17.6)	**<0.001**
Distant metastasis	7 (5.3)	0 (0.0)	7 (41.2)	**<0.001**
Patient death	9 (6.8)	0 (0.0)	9 (52.9)	**<0.001**

Supplementary Table S3. Basal characteristics of pheochromocytoma

Parameters	Total, N=189 (%)
Age (year, mean ±SD)	48.1 ±13.7
Sex	
Male	73 (38.6)
Female	116 (61.4)
Tumor size (cm, mean ±SD)	5.0 ±3.4
Histologic pattern	
Zellballen	165 (87.3)
Non-Zellballen	24 (12.7)
Cellularity	
Low	11 (5.8)
Moderate	162 (85.7)
High	16 (8.5)
Comedo necrosis	
Absent	189 (100.0)
Present	0 (0.0)
Vascular or capsular invasion	
Absent	129 (68.3)
Present	60 (31.7)
Ki-67 labeling index (%)	
<1	139 (73.5)
1-3	38 (20.1)
>3	12 (6.3)
Catecholamine type	
Non-norepinephrine type	154 (81.5)
Norepinephrine type	35 (18.5)
GAPP score	

0-2 (well-differentiated type)	138 (73.0)
3-6 (moderately differentiated type)	50 (26.5)
7-10 (poorly differentiated type)	1 (0.5)
Tumor recurrence	5 (2.6)
Distant metastasis	7 (3.7)
Patient death	11 (5.8)

Supplementary Figure S1. In silico analysis. (A and B) The Gene Expression database of Normal and Tumor Tissues (GENT2) web-accessible database (http://gent2.appex.kr/gent2/) was searched to compare beclin-1 and LC3A expression patterns in adrenal gland tumor and normal tissues. (C) The web-accessible database cBioPortal (http://www.cbioportal.org) was used to evaluate MAP1LC3A gene abnormalities in adrenal gland tumor tissues.

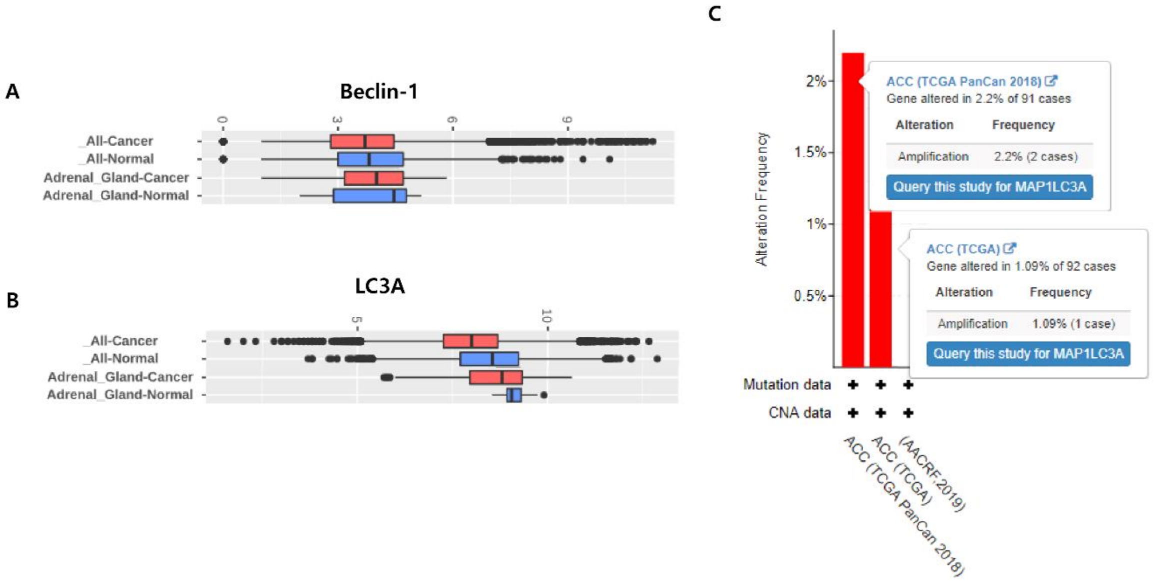

Supplementary Figure S2. Isolated single positive cells (ISPC). In some cases, isolated single positive cells (ISPC) are observed for p62 and LC3B. Strong cytoplasmic expression is detected in one cell.

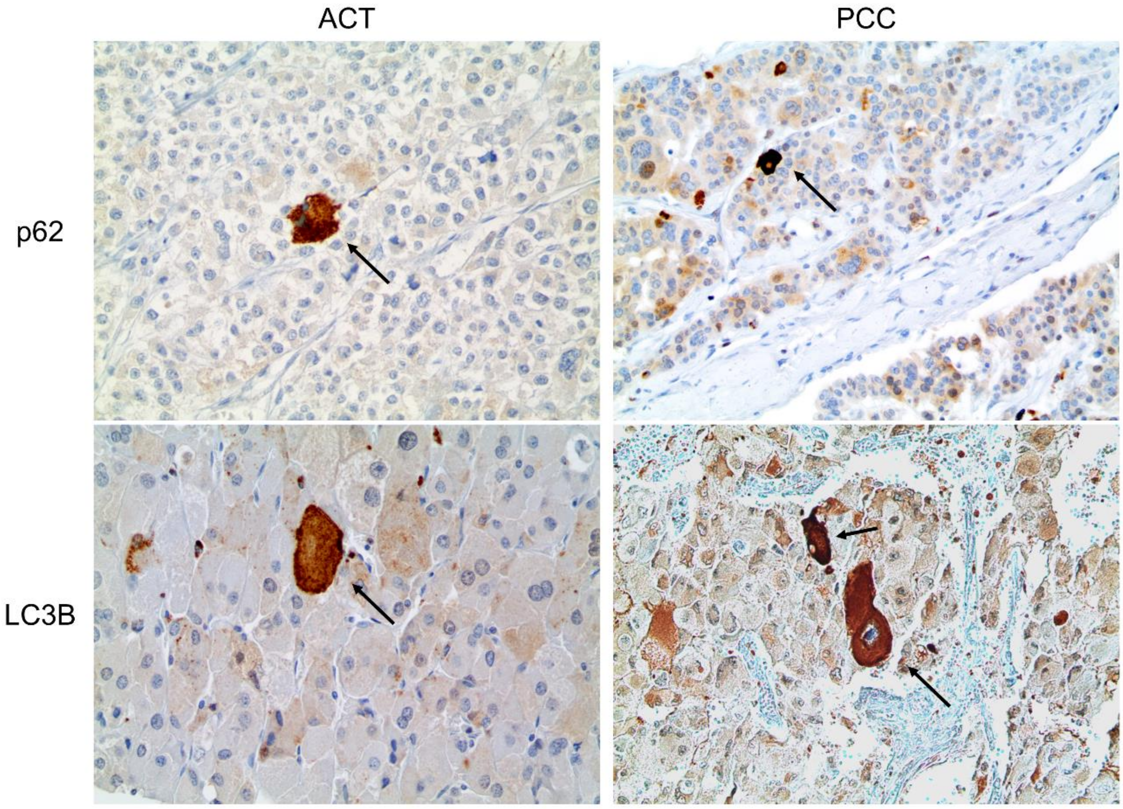

Supplement: Supplementary file 1 [file ijms-22-10490-s001.zip › Supplementary file.pdf]
