# Supplementary figures and images for: Autophagy-Related Proteins Are Differentially Expressed in Adrenal Cortical Tumor/Pheochromocytoma and Associated with Patient Prognosis
Source: Int J Mol Sci. 2021 Sep 28;22(19):10490. doi: 10.3390/ijms221910490 (PMC8508962; doi:10.3390/ijms221910490)

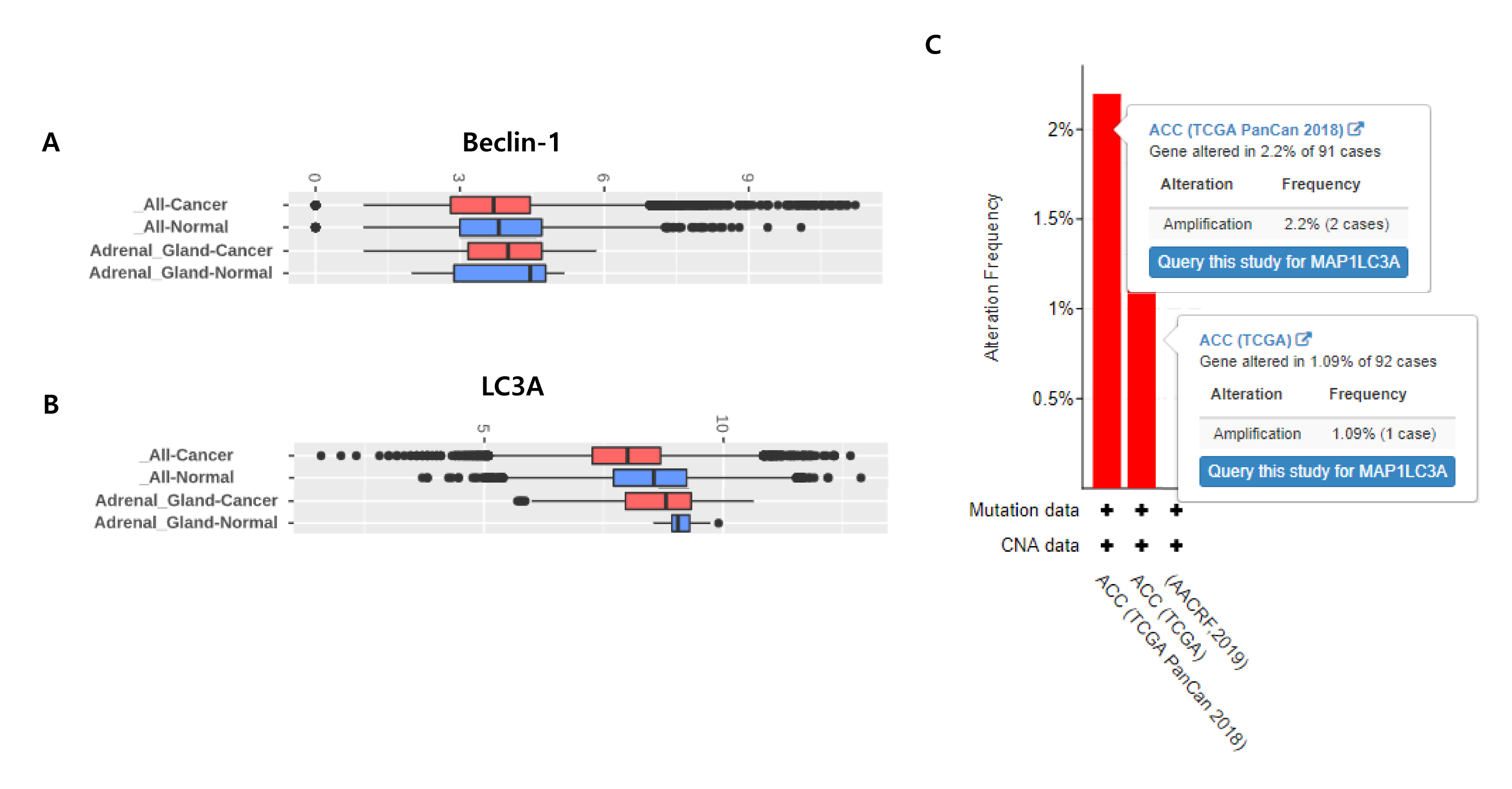

Supplement: Supplementary file 1 [file ijms-22-10490-s001.zip › Supplementary figure S1.tif]

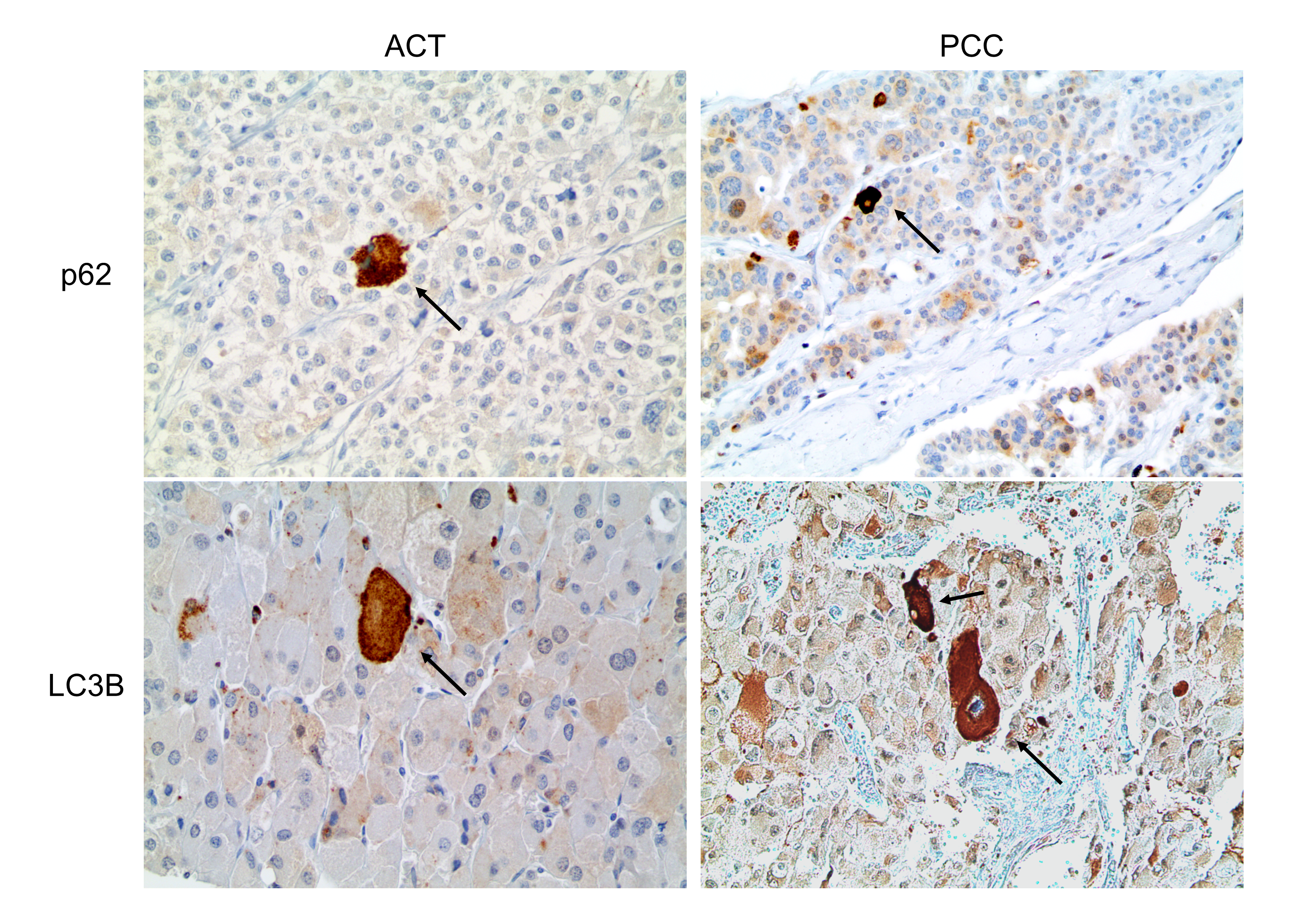

Supplement: Supplementary file 1 [file ijms-22-10490-s001.zip › Supplementary figure S2.tif]
